# The lymphatic system: a pathway for meta-inflammation in permafrost immunity

**DOI:** 10.6026/97320630019886

**Published:** 2023-09-30

**Authors:** Francesco Chiappelli, Lily Fotovat

**Affiliations:** 1 Dental Group of Sherman Oaks, Sherman Oaks, CA 91403; 2 UCLA Center for the Health Sciences, Los Angeles, CA 90095

**Keywords:** Lymphatics, pro-inflammatory cytokines, meta-inflammation, inflamm-aging, permafrost pathogens, pandemic, public health

## Abstract

The lymphatic system is the anatomical substratum of immunity. Lymphatics collect tissue exudates, which contain cell debris, peptides, micronutrients and pathogens, as well as immune naive and memory effector cells from the body tissues and organs into the lymph. Lined by endothelial cells cemented together by tight junctions to ensure their impermeability, lymphatics contain valves that prevent the backward flow of the lymph as it moves forward toward the right and left venous angles, the anatomical site of confluence with the venous blood. Meta-inflammation increases the permeability of lymphatics, rendering the elderly more susceptible to novel and ancient airborne viruses released by melting glaciers and permafrost. Simple public health protocols (e.g., mask-wearing, quarantine) are essential to minimize colliding epidemics/pandemics, and favor permafrost immunity.

## Description:

Greek physician, Hippocrates of Kos, first described nodules about the popliteal area, the anatomically complex and rich in vasculature posterior aspect of the knee joint (Latin, poples: region back of the knee joint). Herophilos of Alexandria later traced the filamentous vessels that connected similar nodules in the abdominal cavity, within which flowed a whitish chyle (Greek, chylos: juice), and which he named lacteals (Latin, lac: milk). Today, the lacteals are known as the lymphatic capillaries wherein flows the lymph (Latin, lympha: water, aqueous humor), a body fluid rich in emulsified fats. Gabrielle Falloppio, who described the Falloppian tubes in the mid 1500's, outlined the course of these lacteals along the mesentery, and Bartolomeo Eustachi, who described the Eustachain tubes of the inner ear in the second half of the 1500's, showed how they pooled into a main vessel along the sternum, the "white vein" (vena alba), today's thoracic duct or left lymphatic duct. By the 1630's Gaspare Aselli's general treaty on the circulation of the lymph (De lactibus sive lacteis venis), published posthumously, detailed the course of the flow of chyle in the complex network of lacteals systemically. Shortly thereafter, Swedish anatomists Thomas Bartholin and Olaus Rudbeck independently reported the intricacies of the lymphatic vasculature; the former, more generalized systemically, the latter, more detailed specifically to the hepatic region. Bartholin was credited for naming this vasculature the lymphatic vessels (or system), an attribution that was forcefully contested by Rudbeck. The lymphatic (or lymphoid) system is the third circulatory system, feeding into, and intimately intertwined with venous and arterial circulation. The lymph circulates through the lymphatic vasculature among and through the primary and the secondary lymphatic organs, collects into the right and the left lymphatic duct, and drains into the systemic venous circulation at the joining of the subclavian and the internal jugular veins, the left and right angulus venosus, proximal to the neurovascular ensheathment of the carotid and axillary sheaths. The left lymphatic duct collects lymph from the lower extremities, and the left side of the body. The right lymphatic duct collects the lymph of the upper right quadrant (right side of head, thorax and upper extremity). In short, the lymphatic network drains, collects and transports extravasated tissue fluid systemically, along with peptides, proteins, micronutrients, and antigens, to the venous and arterial circulatory systems; and it facilitates immune cell migration from the periphery to draining lymph nodes, where engagement with foreign pathogens occurs [1].

Lymphatics connect primary lymphoid organs (i.e., thymus, bone marrow), secondary lymphoid organs (i.e., spleen, lymph nodes) and other ectopic lymphoid organs (i.e., tertiary lymphoid organs that form at sites of sustained inflammation, chronic infection, cancer or otherwise long-term immune activation) [1]. They consist of a simple lining of endothelial cells anchored by tight junctions. They are endowed with a thin layer of smooth muscles and adventitia that bind them to the surrounding tissues, neuromuscular bundles and sheaths. Intra-luminally, lymphatics possess a primary and a secondary valve that ensure the unidirectional flow of lymph without backward reflux. Propulsion of the lymph forward within the lymphatics results from the valvular actions, the lymphatic smooth musculature, and the radiating forces from the striated musculature (i.e., voluntary and involuntary body movement) and mesenteric peristalsis (i.e., involuntary gastrointestinal muscle constriction and relaxation producing forward advancement of intestinal contents) [1].

During aging, a state of chronic systemic inflammation (i.e., meta-inflammation [2], inflamma-aging [3]), develops, which is associated with, if not largely responsible for a variety of aging diseases, including Type-II diabetes and certain neurological pathologies. Meta-inflammatory cytokines impact the micro-anatomy and function of lymph nodes, and lead lymphatics to swell, stretching the endothelial cells, disrupting the tight junctions, and increasing the permeability of the lymphatics. Lymph exudates include physiologic peptides (e.g., neuropeptides, cell debris) and epitopes (i.e., particles from processed pathogenic antigens) activate dendritic cells locally, which secrete pro-inflammatory cytokines (e.g., TNF-α, IL-6, IL-1β, IFN-γ) and nitric oxide, which in turn sustain inflammation, exacerbate swelling of vascular endothelium, and further increase lymphatics permeability [4]. In brief, meta-inflammation causes significant leakage of lymph, and dampening of lymph propulsion. At the cellular level, meta-inflammation cytokines decrease lymphatic endothelial barrier, and blunt intercellular adhesion and intracellular cytoskeletal activation [1-5,6]. Impaired cell migration to and within the nodes, altered structure and organization of nodal zones, and mpaired intercellular interactions lead to decreased survival and function of naïve T cells and, consequentially, diminished B cell maturation and impaired humoral response [6].

At the molecular level, cytokines regulate the expression of the lymphatic endothelial mitogen Vascular Endothelial Growth Factor (VEGF)-C, which binds to the third form of VEGF receptor (VEGFR3) associated with the Flt-4 kinase complex. IL-6 and other meta-inflammation cytokines significantly impair the regulatory modulation of VEGF expression and VEGFR function by lymphatic endothelial cells, and the formation and repair of lymphatics (i.e., lymphangiogenesis) by the Src-FAK-STAT3 cascade [7]. The network efficiency of lymphatics within the central nervous system is dependent upon specialized interactions with glial cell sub-populations (e.g., astroglia), and form a sub-network independent from, yet intertwined with the systemic lymphatic system. The glial-lymphatics, or glymphatics [8], responds similarly as systemic lymphatics to meta-inflammation cytokines, with resulting outcome that may be critical to the etiology and course of Parkinson's disease, dementia, Alzheimer's disease and other neuropathologies [8, 9]. Taken together, data show that cytokines in states of chronic inflammation (i.e., meta-inflammation, inflamm-aging) impair the regulation and function of lymphatics, and favor senescence not only of immunity, but also of its anatomical substratum, the lymphatic system. It follows that meta-inflammation cytokines may significantly contribute to increasing the susceptibility of the elderly to viral epidemics and pandemics, including influenza and CoViD-19, Ebola, Monkeypox, Nipah, Dengue, Zika and other related air-borne, fomite-borne, or vector-transmitted infectious diseases, be they known and current, or novel and ancient pathogens released by melting glaciers and permafrost [10, 11]. Air-borne viruses, from Influenza virus strains (e.g., H1-16N1-9) to Corona virus strains (e.g., MERS, SARS-CoV1-2 and their variants and sub-variants) to Paramyxoviruses (e.g., measles, mumps) and Rhinoviruses (i.e., common cold) are of particular concern not because they can lead to serious epidemics and pandemics (cf., Spanish Flu, CoViD-19) by most immediately penetrating and infecting the nasal and the oral mucosa, and the respiratory tract. They often favor the onset of colliding pandemics [12-13]. As melting glaciers and permafrost release pathogens of various degree of pathogenicity, epidemics and pandemics may unfold as serious public health threats [14] involving:

[1]novel and ancient virus emergence as the eternal ice of glaciers or the permafrost melt

[2]incorporation of these viral species in the autumn polar air circulation (e.g., North Polar Jet stream)

[3]putative onset of epidemics and pandemics in the humid, rainy and cold fall, winter and spring months, which have shorter days and less solar irradiance, thus putatively contributing to vitamin D immune impairment, and possibly intersecting with warmer humid days with consequential accumulation of air-borne viruses particularly vulnerable to certain segments of the human population (e.g., middle-age, aging, elderly),

[4]warmer spring and summer days, including significantly higher solar irradiance that may contribute to ultraviolet immune suppression as one means of pathogenicity amplification, and

[5]viral infection, replication and spread by infected humans, which precipitates population epidemics, decreases herd immunity, and increases the risk of generalized pandemic and rapid global spread, characterized by case number surges (i.e., waves) and multi-year durations, especially when preventive public health measures (e.g., mask-wearing, limited international travel, confinement) are not enforced, or taken too lightly by the population at large.

In conclusion, pro-inflammatory cytokines that initiates and sustain chronic inflammation (i.e., meta-inflammation, inflammation-aging) act in concert to promote immune and lymphatic senescence, impaired viral immunity, and increased permeability of lymphatics to invading novel and ancient viruses. Indeed, when multiple viruses infect simultaneously, an intricate set of pathways, from the host's IFN-γ mediated anti-viral cellular immune surveillance process to sequence-specific gene silencing mechanism guided by double-stranded RNA, converge to contain viral pathogenicity [15,16]. The plethora of cytokines released in meta-inflammation is likely to impair these and related events of viral interference. These lines of evidence demand the attention of public health experts, and of health care specialists (e.g., ENT physicians, dentists) at the frontline of colliding epidemics [12^12^]. Permafrost immunity is directly impacted by sustained simple but effective public health protocols, such mask- and glove wearing, quarantine designed to limit and contain viral infections.
